# Supplementary material for: Treatment outcome and associated factors among adult patients with pulmonary tuberculosis in selected health centers in Addis Ababa Ethiopia
Source: PLoS One. 2023 Oct 5;18(10):e0292218. doi: 10.1371/journal.pone.0292218 (PMC10553344; doi:10.1371/journal.pone.0292218)
Supplement: Attachment — Submitted filename: Review assignment for PONE-D-23-04424.docx [file pone.0292218.s001.docx]

Review assignment for PONE-D-23-04424

This article talk about the research of the factors associated with the therapeutic success in the tuberculosis treatment in Addis Abeba and around.

In the introduction according to the annual FDRE MOH the success rate in Addis Abeba was 90% in accordance with international requirements but not well understood the inconsistencies because in the same country the reasons can be different by city because different environment.

It would be better to explain the inconsistencies that require this study.

If the term therapeutic success is the desired goal, there must be consistency in the writing to facilitate reading

It will also be necessary to harmonize the font

For the sampling I did not understand the technique of the proportional allocation. This proportionality is a function of the number of patients followed or the geolocalization
