# Supplementary material for: Treatment outcome and associated factors among adult patients with pulmonary tuberculosis in selected health centers in Addis Ababa Ethiopia
Source: PLoS One. 2023 Oct 5;18(10):e0292218. doi: 10.1371/journal.pone.0292218 (PMC10553344; doi:10.1371/journal.pone.0292218)
Supplement: Attachment — Submitted filename: Response to Reviewers.docx [file pone.0292218.s002.docx]

**Dear	Chief-editor and Reviewers,**

# Thank you for taking your precious time reviewing our manuscript: ID PONE-D-23-04424 **“*Treatment Outcome and associated factors among Adult Patients with Pulmonary Tuberculosis in Selected Health Centers in Addis Ababa Ethiopia”.***

# We have received the reviewer comments from Elsevier’s Author Hub. All comments focused on the instrument section and we have addressed the comments as follows:

**Reviewer's comments and their response**

**Comments:** Ensure that your PLOS ONE's style requirements

**Response** Manuscript edited to meets PLOS ONE's style requirements

**Comment: :**Include the relevant URLs, DOIs, or accession numbers within your revised cover letter

Access URLs included in the data availability section and the revised cover letter

**Comment:** Include a separate caption for figure in your manuscript

**Response:** Figure caption is included as recommended

**Comment:** Give number ‘1’for all authors as they all have the same affiliation

**Response:** Changes made as suggested by the editors

**Comment:** Is this study retrospective cross-sectional study or retrospective cohort study?

**Response:** The study design is retrospective cohort study, since the analysis involves comparison of groups

**Comment:** Define or describe ‘standard anti TB drug regimen’

**Response:** Standard anti TB drug regimen used by studied patients stated as suggested

**Comment:** Sampling technique and procedure’ need revision: how many sub-cities are there in Addis Ababa?

**Response:** Revised and missing components added as per the suggestion

**Comment:** State the correct that office that is responsible issue ethical approval in the institution.

**Response:** Ethical approval is issued at College level and the section is revised as the College of Health Sciences.

**Comment:** The English language used in the submission must be edited.

**Response:** The English language edited in consultation with language experts.

**Comment:** Flow of the article must be corrected.

**Response:** Article flow corrected as per the suggestion.

**Comment:** Material and methods section abstract is over long

**Response:** We decided to keep this section since there are no word count recommendations for the journal, and we believed the components are important.

**Comment:** The introduction section is also very long. Please shorten this section according to the main objective of your study.

**Response:** Revised as suggested.

**Comment:** You didn’t include the patients below 18 years.

**Response:** Changes has been made to the title since all participants were adult patients.

**Comment:** You studied a limited number of potential risk factors in your study.

**Response:** We were limited to fewer numbers of risk factors for the fact that some of the variables were not recorded in the patient records. Therefore, we have included statement to the study limitation section.

**Comment:** Please extend your discussion with the results of similar studies.

**Response:** Efforts made to make **c**hanges in the section

.
