# Supplementary material for: Treatment outcome and associated factors among adult patients with pulmonary tuberculosis in selected health centers in Addis Ababa Ethiopia
Source: PLoS One. 2023 Oct 5;18(10):e0292218. doi: 10.1371/journal.pone.0292218 (PMC10553344; doi:10.1371/journal.pone.0292218)
Supplement: Attachment — Submitted filename: Review assignment V2VD for PONE-D-23-04424.docx [file pone.0292218.s003.docx]

Review assignment for PONE-D-23-04424

This article talk about the research of the factors associated with the therapeutic success in the tuberculosis treatment in Addis Abeba and around.

I didn't quite understand the term proportionality in this sentence.

« .Within the four Sub-cities, there were twenty health

105 centers and one third (six), health centers were selected proportionally from each sub city

106 according to the number of health centers in each. »

proportional to what?

One of your selection criteria was to receive a selection of centers with a number of people to consult greater than .........

« A structured checklist adopted from previous studies on a similar topic was used to extract data

111 (8) »

The information on the checklist is insuffisant. checklist is validated ? have you done a pretest?

The Authors say that it has been used in several studies but there is only one reference. this checklist should be part of your limits because the measuring tool is not necessarily the same as the studies you are comparing the results of your study.

About the limits of the study

The question is: can this study be generalized to the whole country?

The Authors must add the criteria of information bias with the checklist and perhaps selection bias, as those who were very ill may not have been able to visit the centers.
