# Supplementary material for: Treatment outcome and associated factors among adult patients with pulmonary tuberculosis in selected health centers in Addis Ababa Ethiopia
Source: PLoS One. 2023 Oct 5;18(10):e0292218. doi: 10.1371/journal.pone.0292218 (PMC10553344; doi:10.1371/journal.pone.0292218)
Supplement: Attachment — Submitted filename: Response to review comments PTB treatment outcome.docx [file pone.0292218.s004.docx]

**Dear	Chief-editor and Reviewers,**

Thank you for taking your precious time reviewing our manuscript: ID PONE-D-23-04424 **“*Treatment Outcome and associated factors among Adult Patients with Pulmonary Tuberculosis in Selected Health Centers in Addis Ababa Ethiopia”.***

# We have received the reviewer comments from Elsevier’s Author Hub. All comments focused on the instrument section and we have addressed the comments as follows:

**Response to review comments:**

| **Additional Information:** | | | |
| --- | --- | --- | --- |
| S/No | **Comment** | **Response** | |
| 1 | Include the sub-heading ‘**Introduction**’ in the abstract | Sub-heading ‘**Introduction**’ included in the abstract section | |
| 2 | In the manuscript, both retrospective cross-sectional and cohort study designs are mentioned. The appropriate study design needs to be used consistently thorough out the manuscript. | The study design is revised as retrospective cross-sectional study design thorough out the document as recommended. | |
| 3 | • Paraphrase descriptions in abstract line #35 “Convenient sampling techniques were employed to review patient…” convenient sampling technique is to be used to select study participants | The statement convenient sampling techniques were employed to review patient charts, paraphrased as suggested in the comment | |
| 4 | Omit “Background” from line #53 | Sub-heading “Background” omitted from the introduction section. | |
| 5 | Line #75 “….outcomes was 20.4, and 19.8 respectively” Are these values in % or frequency? | The outcome values 20.4, and 19.8 are percent and indicated in the mentioned values. | |
| 6 | Line # 123 in a statement “was obtained from Research Ethics Committee of Addis Ababa University..” nomenclature ‘Research Ethics Committee needs to be checked out unless it is changed; the correct name is ‘Institutional Review Board’ check and amends wherever it applies | Nomenclature of the Ethics approval body is revised as “**Institutional review board** **of Collage of Health Science in Addis Ababa University**”. | |
| 7 | Consider including sample size determination and how proportional allocation was performed? | Sample size calculation included as indicated in the appropriate section | |
| 8 | Include all abbreviations used in the tables as footnotes in their expanded form | Abbreviations used within tables are indicated as footnotes in their expanded form as recommended | |
| 9 | Table 1. Are age categories in years? Include unit for weight | Unit of measurement for age included as “e in years”. | |
| 10 | I suggest using ‘multivariable analysis’ instead of ‘multivariate analysis | Multivariate analysis method is replaced as “multivariable” as recommended by the editor. | |
|  | **Reviewer** | | |
| 1 | I didn't quite understand the term proportionality in this sentence.  « .Within the four Sub-cities, there were twenty health  105 centers and one third (six), health centers were selected proportionally from each sub city  106 according to the number of health centers in each. »  proportional to what?  One of your selection criteria was to receive a selection of centers with a number of people to consult greater than ......... | | This statement is revised as “Among the ten Sub-cities in Addis Ababa, four sub-cities which include, Nifas silk, Lideta, Gullele and Yeka were selected using simple random sampling method. There were twenty health centers within the four Sub-cities, and (six), health centers were in the same manner the Sub-cities selected”. |
| 2. | A structured checklist adopted from previous studies on a similar topic was used to extract data (8).  The information on the checklist is insufficient. Checklist is validated ? have you done a pretest? | | We have used data from the existing cited sources (5, 8). we assumed these articles to be validated earlier, however the checklist was pretested in a setting other that the selected study settings. |
| 3. | The Authors say that it has been used in several studies but there is only one reference. This checklist should be part of your limits because the measuring tool is not necessarily the same as the studies you are comparing the results of your study. | | This is revised and included more citation. |
| 4 | Can this study be generalized to the whole country? | | The response for this question is already reflected in the limitation section, stating “ the findings may not be generalized beyond the study settings. |
| . 5 | The Authors must add the criteria of information bias with the checklist and perhaps selection bias, as those who were very ill may not have been able to visit the centers | | This is also included in the limitation of the study. |
