# Supplementary material for: Treatment outcome and associated factors among adult patients with pulmonary tuberculosis in selected health centers in Addis Ababa Ethiopia
Source: PLoS One. 2023 Oct 5;18(10):e0292218. doi: 10.1371/journal.pone.0292218 (PMC10553344; doi:10.1371/journal.pone.0292218)
Supplement: Attachment — Submitted filename: Response to the editor.docx [file pone.0292218.s005.docx]

**Dear	Chief-editor and Reviewers,**

Thank you for taking your precious time reviewing our manuscript: ID PONE-D-23-04424 **“*Treatment Outcome and associated factors among Adult Patients with Pulmonary Tuberculosis in Selected Health Centers in Addis Ababa Ethiopia”.***

# We have received the reviewer comments from Elsevier’s Author Hub. All comments focused on the instrument section and we have addressed the comments as follows:

**Response to review comments:**

| **Additional Information:** | | |
| --- | --- | --- |
| S/No | **Comment** | **Response** |
| 1 | Upload a copy of Figure 1 which you refer to in your text on page 9 | Figure 1 uploaded |
